# Parecoxib inhibits glioblastoma cell proliferation, migration and invasion by upregulating miRNA-29c

**DOI:** 10.1242/bio.021410

**Published:** 2016-11-28

**Authors:** Lin-Yong Li, Jie Xiao, Qiang Liu, Kun Xia

**Affiliations:** 1 The State Key Laboratory of Medical Genetics, School of Life Sciences, Central South University, Changsha, Hunan, China, 410078; 2 Department of Neurosurgery, The Third Xiangya Hospital of Central South University, Changsha, Hunan, China, 410013; 3 Department of Emergency, The Third Xiangya Hospital of Central South University, Changsha, Hunan, China, 410013

**Keywords:** Glioblastoma, Cyclooxygenase-2, Parecoxib, miRNA-29c, Proliferation, Invasion

## Abstract

Glioblastoma (GBM) is one of the most lethal brain cancers worldwide, and there is an urgent need for development of novel therapeutic approaches. Parecoxib is a well-known cyclooxygenase-2 (COX-2) inhibitor, and had already been developed for postoperative analgesia with high efficacy and low adverse reaction. A recent study has suggested that parecoxib potently enhances immunotherapeutic efficacy of GBM, but its effects on GBM growth, migration and invasion have not previously been studied. In the present study, MTT [3-(4,5-dimethylthiazol-2-yl)-2,5-diphenyltetrazolium bromide] and BrdU (5-bromo-2-deoxyuridine) incorporation assays were used to evaluate the cell proliferation of GBM cells. Wound-healing and transwell assays were preformed to analyze GBM cell migration and invasion, respectively. The results suggested that parecoxib inhibits cell proliferation, migration and invasion of GBM cells in a dose-dependent manner. RT-qPCR (real-time quantitative PCR) analysis demonstrated that miRNA-29c can be significantly induced by parecoxib. Furthermore, our data suggests that a miRNA-29c inhibitor can significantly attenuate parecoxib's effect on proliferation, migration and invasion of GBM. In conclusion, the present study suggests that parecoxib inhibits GBM cell proliferation, migration and invasion by upregulating miRNA-29c.

## Abstract (precis)

**Summary:** The study indicated that parecoxib may be a potential therapeutic approach to treat GBM by up-regulating miRNA-29c and subsequently inhibiting proliferation, migration and invasion of GBM.

## INTRODUCTION

Glioblastoma (GBM) is a grade IV glioma classified by the World Health Organization (WHO), which is one of the most lethal and aggressive brain cancers, and accounts for 15% of brain malignancies (Young et al., 2015). The typically treatment for GBM involves surgery, chemotherapy, radiotherapy or combination therapy. Although the therapies for GBM have largely improved in the past few decades, the survival rate of patients with GBM is still low, as less than approximately 5% of patient survive more than five years (Gallego, 2015). Therefore, there is an urgent need to explore and develop new therapeutic approaches for prevention and treatment for the deadly malignancy.

Frequently, overexpression of cyclooxygenase-2 (COX-2) had been found in several types of tumor, including breast cancer (Regulski et al., 2015) and glioblastoma (Onguru et al., 2008), and implicated in inflammation and tumorigenesis, indicating that inhibition of COX-2 may exhibit a potential anticancer effect. Accumulating data indicated that COX-2 inhibitors, the non-steroidal anti-inflammatory drugs, are promising chemoprevention and chemotherapeutic agents that may protect against breast, brain, lung, esophageal, colon, and oral tumors (Dang et al., 2002; Menter, 2002). Of the COX-2 inhibitors, parecoxib is one of the most well-known COX-2 selective inhibitors, which had been developed as a highly efficient postoperative analgesia drug with low adverse reaction, and parecoxib treatment was shown to exert a potent anticancer role in multiple human cancers, including colorectal cancer (Zagani et al., 2009; Xiong et al., 2015), esophageal adenocarcinoma (Santander et al., 2012). It is worth noting that parecoxib treatment was capable of enhancing immunotherapy of brain tumors. A recent study indicated that intratumoral COX-2 inhibition by using parecoxib or valdecoxib potentiates GM-CSF immunotherapy against established mouse GL261 brain tumors (Eberstål et al., 2014). Another study also found that inhibition of COX-2 by using parecoxib potently enhances immunotherapeutic efficacy of GBM (81% survival), compared to immunotherapy alone (19% survival) (Eberstål et al., 2012). Importantly, parecoxib was able to rapidly penetrate the blood–brain barriers, thereby making parecoxib convenient for treatment of brain tumors, such as GBM. However, the anticancer effect of parecoxib on GBM has not been fully studied before now.

MicroRNAs (miRNAs) are a class of 21-25 nucleotide small noncoding RNAs that post-transcriptionally downregulate expression of various genes via binding to the 3′ untranslated region (UTR) of mRNA of the target gene, leading to translational suppression or mRNA cleavage (Bartel, 2004). Accumulating studies have indicated that miRNAs play a critical role in controlling a wide range of cellular processes, including cell differentiation, cell proliferation, death and development (Ambros, 2004). Aberrant expression of miRNAs is closely associated with carcinogenesis, and cancer-related miRNA may play tumor suppressive or oncogenic roles (Calin and Croce, 2006). miRNA-29c is an important tumor suppressor miRNA in various human cancers (Lu et al., 2016), and may act as a promising therapeutic agent against human cancer. In GBM, miRNA-29c is a potential prognostic marker, as its expression negatively correlates with glioma grade (Wang et al., 2013). Moreover, miRNA-29c was significantly downregulated in glioma cells and tissues, and inhibits glioma cell proliferation, migration, invasion and angiogenesis via targeting MMP-2 and downregulating VEGF (Fan et al., 2013). Interestingly, selective COX-2 inhibitors have potential for treatment of gastric cancer via an increase in miRNA-29c (Saito et al., 2013). However, the effect of parecoxib on miRNA-29c in GBM remains to be elucidated.

To investigate the anticancer role of parecoxib in GBM, we treated GBM cells with parecoxib and detected cell proliferation, migration and invasion. The results suggested that treatment with parecoxib decreases the cell proliferation, migration and invasion ability of GBM cells in a dose-dependent manner. Further studies identified that miRNA-29c was significantly induced by parecoxib, and addition of a miRNA-29c inhibitor can significantly attenuate parecoxib's chemotherapeutic efficacy. These finding suggest that parecoxib treatment can inhibit GBM cell proliferation, migration and invasion via upregulating miRNA-29c.

## RESULTS

### Parecoxib inhibits the cell growth of GBM cells

**Fig. 1.**
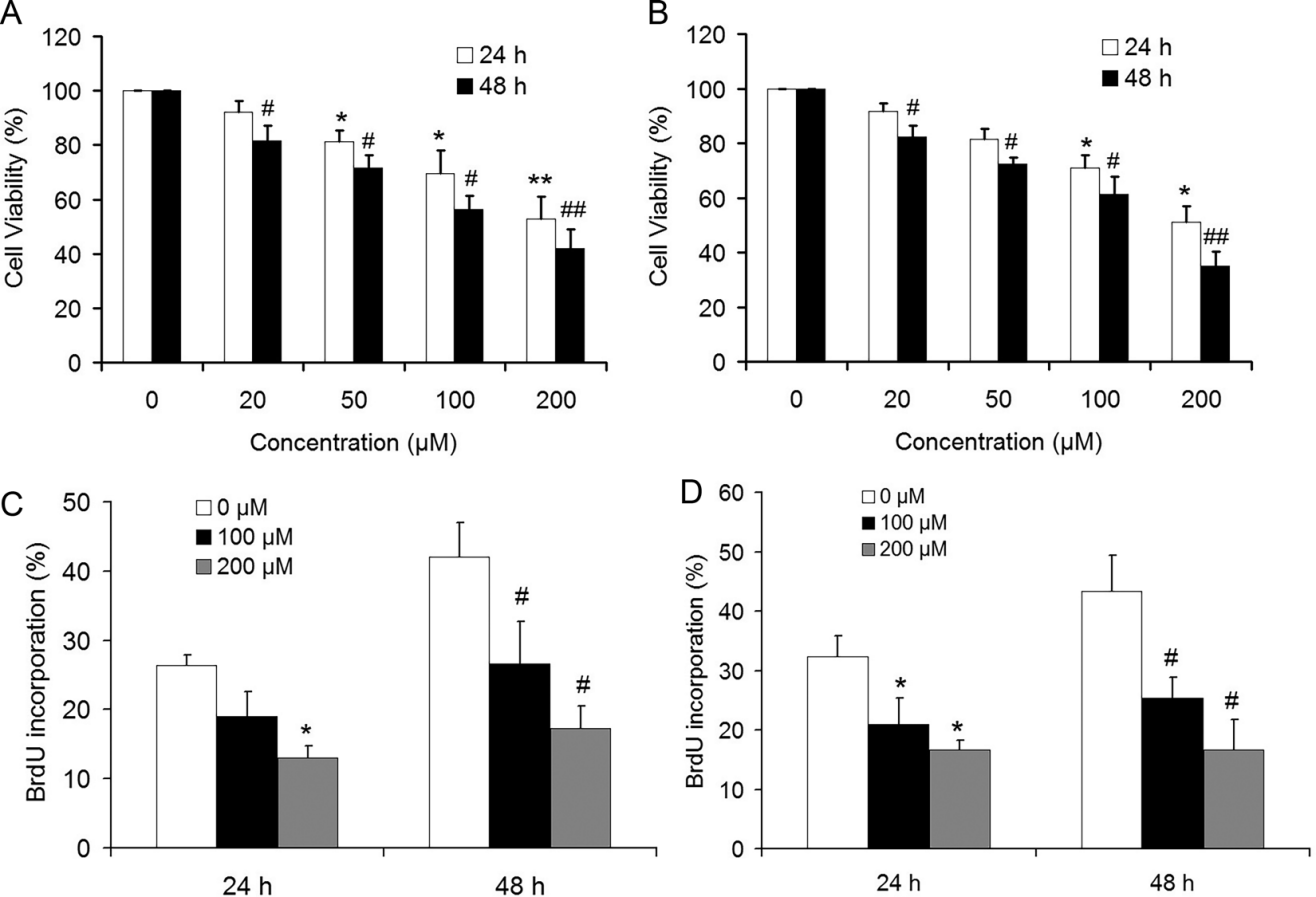
**Parecoxib inhibits cell proliferation of GBM cells.** (A) The relative cell viability of U251 cells was determined by MTT assay. U251 cells were treated with 0, 20, 50, 100, and 200 μM of parecoxib for 24 and 48 h. Mean±s.d., *n*=3, *P<0.05, **P<0.01 compared with 0 μM of parecoxib at 24 h; #*P*<0.05, ##*P*<0.01 compared with 0 μM of parecoxib at 48 h. (B) The relative cell viability of U343 cells was determined by MTT assay. U343 cells were treated with 0, 20, 50, 100, and 200 μM of parecoxib for 24 and 48 h. Mean±s.d., *n*=3, *P<0.05 compared with 0 μM of parecoxib at 24 h; #*P*<0.05, ##*P*<0.01 compared with 0 μM of parecoxib at 48 h. (C) The BrdU incorporation of U251 cells was determined by BrdU assay. U251 cells were treated with 0, 100, and 200 μM of parecoxib for 24 and 48 h. Mean±s.d., *n*=3, *P<0.05 compared with 0 μM of parecoxib at 24 h; #*P*<0.05 compared with 0 μM of parecoxib at 48 h. (D) The BrdU incorporation of U343 cells was determined by BrdU assay. U343 cells were treated with 0, 100, and 200 μM of parecoxib for 24 and 48 h. Mean±s.d., *n*=3, *P<0.05 compared with 0 μM of parecoxib at 24 h; #*P*<0.05 compared with 0 μM of parecoxib at 48 h.

To investigate the effect of parecoxib on the cell growth of GBM cells, U251 and U343 cells were exposed to the increased parecoxib concentrations (0, 20, 50, 100 and 200 μM) for 24 and 48 h. The results of MTT assays showed that the growth rate of U251 and U343 cells was significantly decreased in a dose-dependent manner (Fig. 1A,B). Moreover, the BrdU assays also showed similar results, as 100 and 200 μM of parecoxib treatment resulted in a slower BrdU incorporation rate of GBM cells including U251 and U343 cells compared with control (Fig. 1C,D). These results indicated that parecoxib inhibits the cell proliferation of GBM cells.

### Parecoxib inhibits the migration ability of GBM cells

**Fig. 2.**
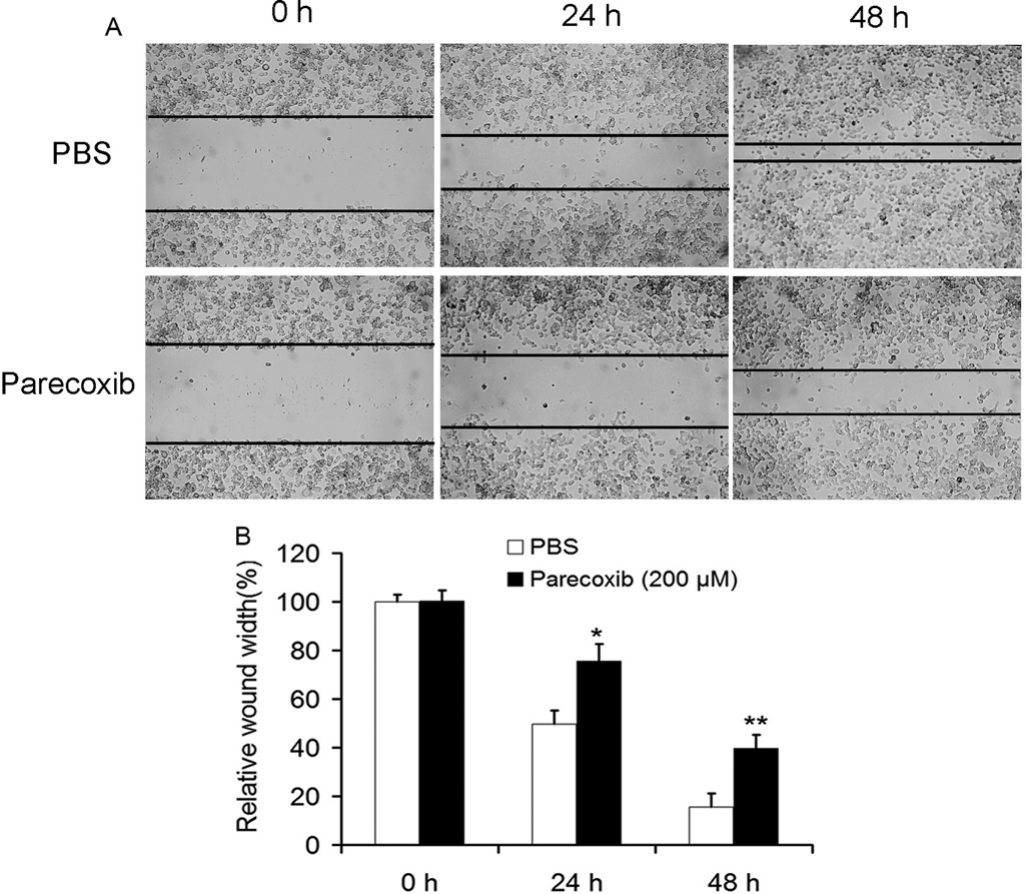
**Parecoxib inhibits cell migration of GBM cells.** (A) The cell migration of U343 cells was assessed by wound-healing assay. U343 cells were scratched by sterile 10 μl pipette tip, followed by treatment with PBS or 200 μM of parecoxib for 24 and 48 h. After scratch, U343 cells were photographed at 200× magnification. (B) The wound width was measured at 0, 24 and 48 h. Parecoxib suppresses healing of the scratched cell. Mean±s.d., *n*=3, *P<0.05 compared with PBS treatment at 24 h; **P<0.01 compared with PBS treatment at 48 h.

To investigate the effect of parecoxib on the migration ability of GBM cells, U343 cells were treated with parecoxib for 24 and 48 h, followed by the scratch wound healing motility assay. The results showed that U343 cells treated with 200 μM of parecoxib for 24 and 48 h migrated significantly slower than PBS-treated control cells (Fig. 2A,B), suggesting that parecoxib could significantly decrease migratory ability.

### Parecoxib inhibits the invasive ability of GBM cells

**Fig. 3.**
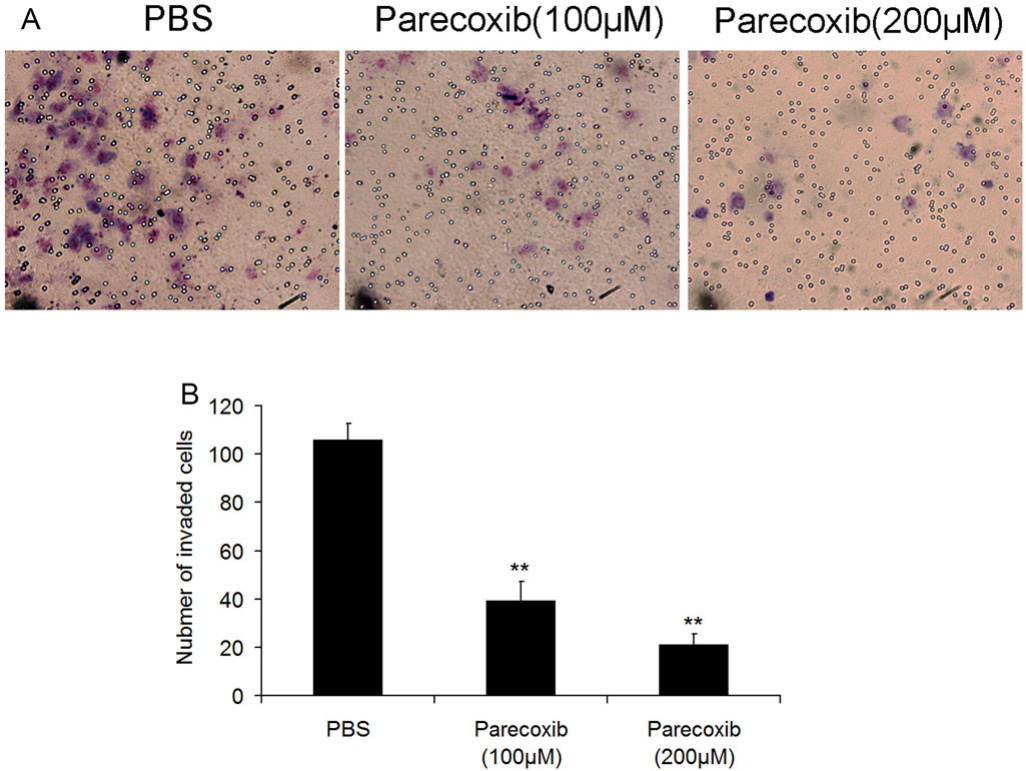
**Parecoxib inhibits cell invasion of GBM cells.** (A) The cell invasion of U343 cells was detected by transwell assay. U343 cells were seeded in the chamber, followed by treatment with 100 μM or 200 μM of parecoxib for 48 h. After staining with Crystal Violet, U343 cells were photographed at 400× magnification. (B) Parecoxib significantly suppresses invasion of U343 cells. Mean±s.d., *n*=3, **P<0.01 compared with PBS treatment at 48 h.

The effect of parecoxib on the invasive ability of U343 cells was further investigated by using a Transwell invasion assay. Compared with the control, the number of invasive GBM cells incubated with 100 μM and 200 μM parecoxib was decreased significantly (Fig. 3A,B), indicating that parecoxib could significantly decrease the invasion abilities of GBM cells.

### Parecoxib upregulates miRNA-29c and downregualtes its target genes in GBM cells

**Fig. 4.**
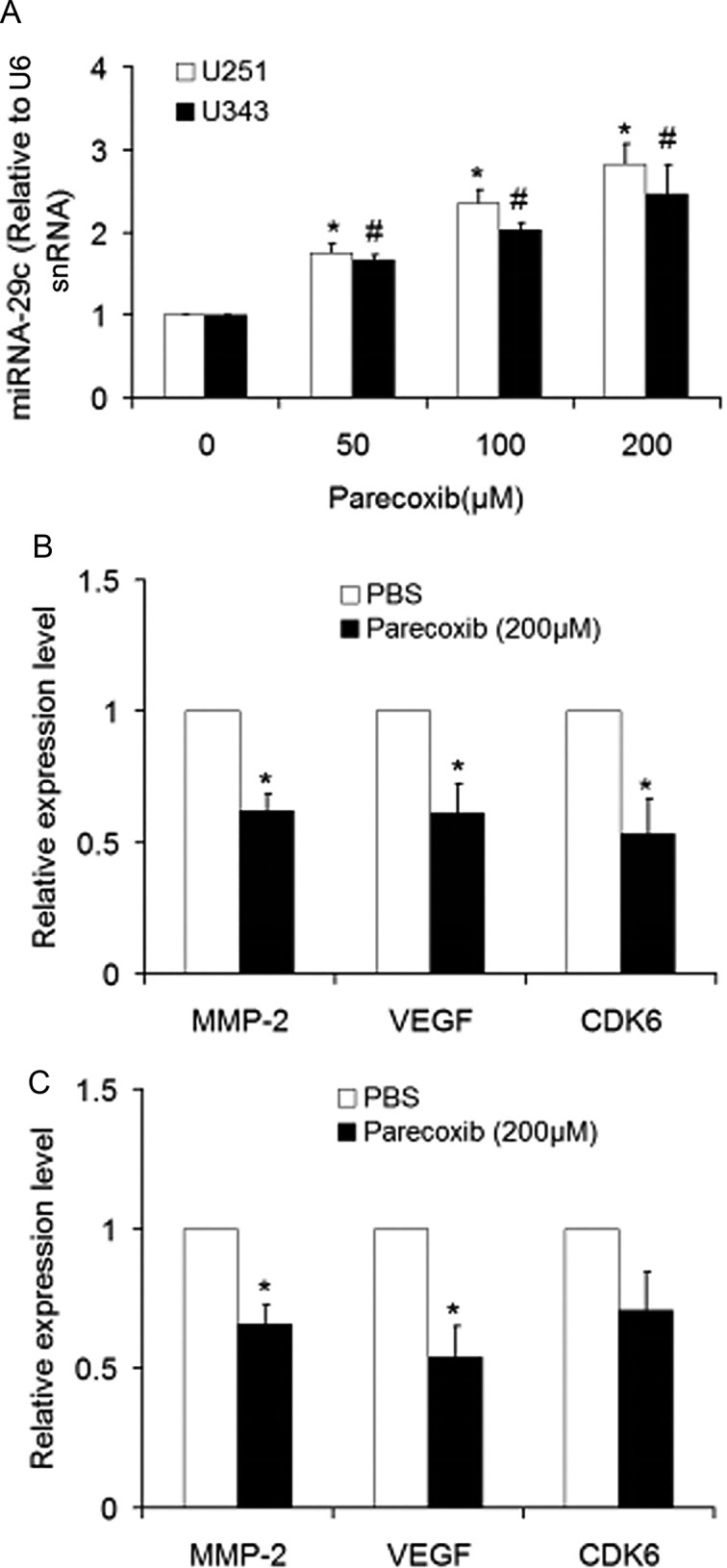
**Parecoxib upregulates miRNA-29c and downregulates its target genes in GBM cells.** (A) The level of miRNA-29c was determined by RT-qPCR. U251 and U343 cells were treated with 0, 50, 100 or 200 μM of parecoxib for 24 h. Mean±s.d., *n*=3, *P<0.05 compared with U251 cells treated with 0 μM of parecoxib at 24 h; #*P*<0.05 compared with U343 cells treated with 0 μM of parecoxib at 24 h. (B) mRNA levels of miRNA-29c target genes including MMP-2, VEGF and CDK6 in U251 cells were determined by RT-qPCR. U251 cells were treated with 0 or 200 μM of parecoxib for 24 h. Mean±s.d., *n*=3, *P<0.05 compared with U251 cells treated with 0 μM of parecoxib (PBS) at 24 h. (C) mRNA levels of miRNA-29c target genes including MMP-2, VEGF and CDK6 in U343 cells were determined by RT-qPCR. U343 cells were treated with 0 or 200 μM of parecoxib for 24 h. Mean±s.d., *n*=3, *P<0.05 compared with U343 cells treated with 0 μM of parecoxib (PBS) at 24 h.

miRNA-29c is an important tumor suppressor miRNA, which is downregulated in GBM (Wang et al., 2013; Fan et al., 2013) and can be induced by the COX-2 inhibitor celecoxib (Saito et al., 2013). The effect of parecoxib on miRNA-29c expression was thus determined by using real-time quantitative PCR (RT-qPCR). The results showed that parecoxib could significantly increase miRNA-29c expression in U251 and U343 cells in a dose-dependent manner (Fig. 4A). Previous studies suggested that miRNA-29c is able to downregulate MMP-2 (Fan et al., 2013), VEGF (Fan et al., 2013), and CDK6 (Wang et al., 2013). In this study, we found that treatment with 200 μM of parecoxib in U251 cells significantly results in the decrease of MMP-2, VEGF and CDK6 mRNA levels (Fig. 4B). A similar result was also observed in U343 cells (Fig. 4C). These data indicate that parecoxib can inhibit cell proliferation and invasion by downregulating miRNA-29c and upregulating its target genes.

### miRNA-29c inhibitor attenuates the inhibitory effect of parecoxib on cell proliferation of GBM cells

**Fig. 5.**
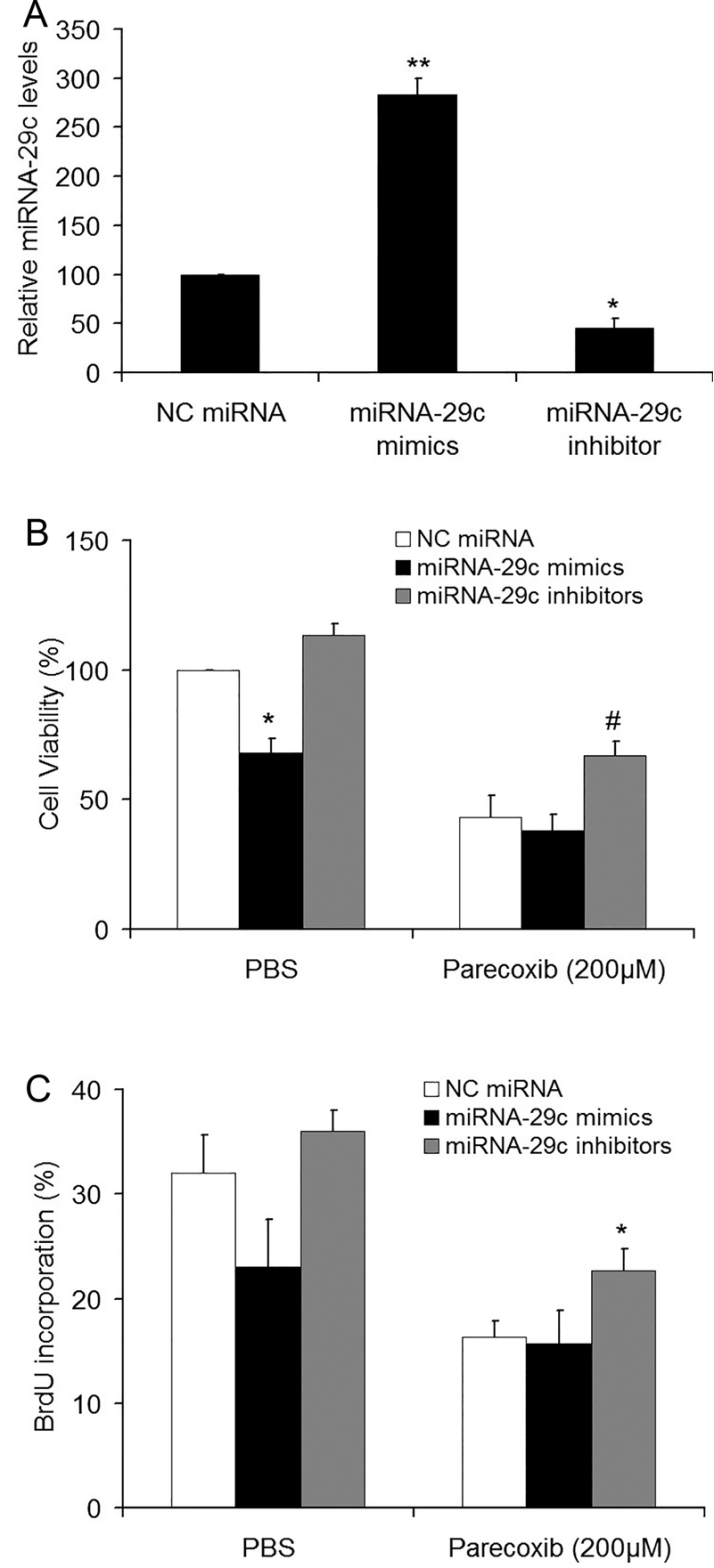
**miRNA-29c inhibitors attenuated the effect of parecoxib on cell proliferation of GBM cells.** (A) miRNA-29c level was determined by RT-qPCR. U343 cells were transfected with NC miRNA, miRNA-29c mimics or miRNA-29c inhibitors for 24 h. Mean±s.d., *n*=3, *P<0.05, **P<0.01 compared with U343 cells transfected with NC miRNA. (B) The relative cell viability of U343 cells was determined by MTT assay. U343 cells were transfected with NC miRNA, miRNA-29c mimics or miRNA-29c inhibitors for 24 h, followed by treatment with 0 or 200 μM of parecoxib for another 24 h. Mean±s.d., *n*=3, *P<0.05 compared with cells treated with NC miRNA and PBS; #*P*<0.05 compared with cells treated with NC miRNA and 200 μM of parecoxib. (C) The BrdU incorporation of U343 cells was determined by BrdU assay. U343 cells were transfected with NC miRNA, miRNA-29c mimics or miRNA-29c inhibitors for 24 h, followed by treatment with 0 or 200 μM of parecoxib for another 24 h. Mean±s.d., *n*=3, *P<0.05 compared with cells treated with NC miRNA and 200 μM of parecoxib.

miRNA-29c expression was induced by parecoxib, indicating that miRNA-29c may mediate the chemotherapeutic efficacy of parecoxib. We thus investigated the effect of miRNA-29c on parecoxib-inhibited cell proliferation. The specific miRNA-29c mimics, miRNA-29c inhibitors or scramble miRNA were transiently transfected into U343 cells for 24 h, followed by treatment with parecoxib for another 24 h. RT-qPCR analysis showed that the expression of miRNA-29c was significantly increased and reduced after transfection of miRNA-29c mimics and inhibitors, respectively, in comparison with scramble miRNA (Fig. 5A). The MTT assays showed that overexpression of miRNA-29c mimics significantly inhibited the cell viability of GBM cells, and a combination of miRNA-29c mimics and parecoxib could not further decrease the cell viability compared to parecoxib treatment alone. However, the growth rates in the miRNA-29c inhibitors+parecoxib group were significantly higher than the NC miRNA+parecoxib group (Fig. 5B). Similar results were also observed in BrdU assays (Fig. 5C).

### miRNA-29c inhibitors attenuate the inhibitory effect of parecoxib on cell migration of GBM cells

**Fig. 6.**
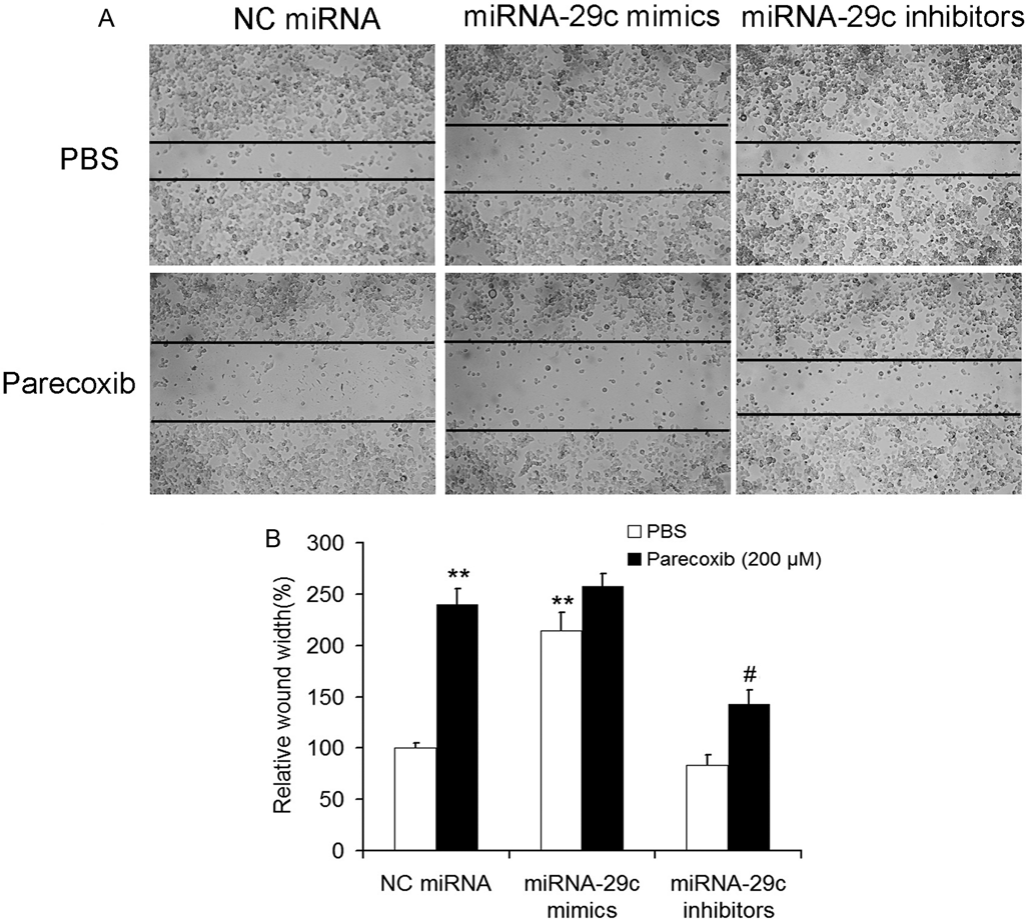
**miRNA-29c inhibitors attenuated the inhibitory effect of parecoxib on cell migration of GBM cells.** (A) The cell migration of U343 cells was assessed by wound-healing assay. U343 cells were transfected with miRNA-29c mimics or inhibitors for 24 h. After transfection, the cells were scratched with a sterile 10 μl pipette tip, followed by treatment with PBS or 200 μM of parecoxib for another 24 h. U343 cells were photographed at 200× magnification after treatment. (B) miRNA-29c inhibitors attenuated the effect of parecoxib on scratched cell healing. Mean±s.d., *n*=3, **P<0.01 compared with U343 cells treated with NC miRNA and PBS; #*P*<0.05 compared with U343 cells treated with NC miRNA and 200 μM of parecoxib.

The effects of miRNA-29c on parecoxib inhibited migration were also determined by using a scratch wound-healing motility assay in U343 cells. Although the migratory abilities of U343 cells in the miRNA-29c mimics+parecoxib group were almost the same as the NC miRNA+parecoxib group, the migratory abilities in the miRNA-29c inhibitors+parecoxib group were significantly lower than the NC miRNA+parecoxib group (Fig. 6A,B).

### miRNA-29c inhibitors attenuate the inhibitory effect of parecoxib on cell invasion of GBM cells

**Fig. 7.**
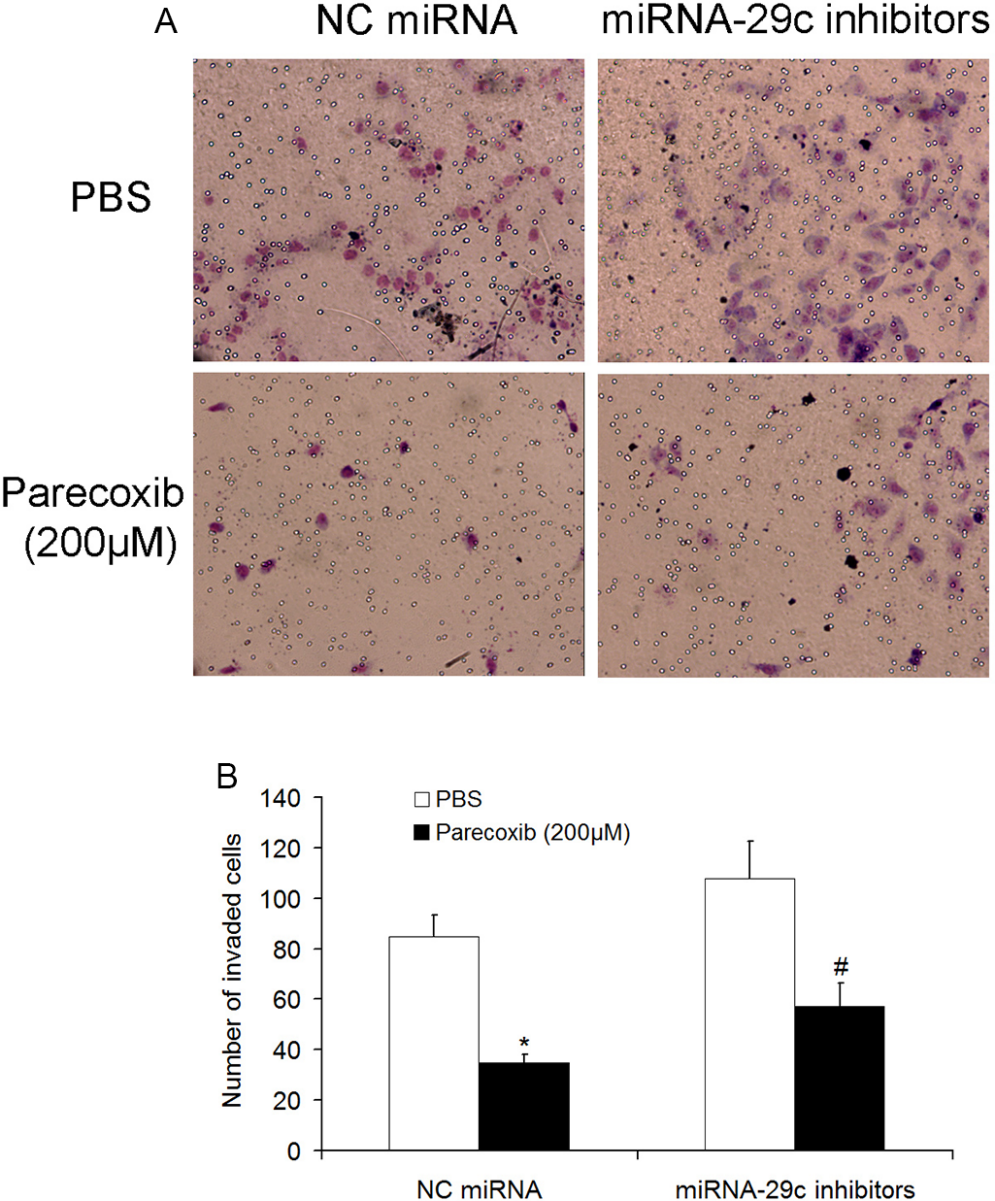
**miRNA-29c inhibitors attenuated the inhibitory effect of parecoxib on cell invasion of GBM cells.** (A) The invasion of U343 cells was detected by transwell assay. U343 cells were transfected with NC miRNA or miRNA-29c inhibitors for 24 h, subsequently seeded in the chamber, and followed by treatment with PBS or 200 μM of parecoxib for another 24 h. After staining with Crystal Violet, U343 cells were photographed at 400× magnification. (B) miRNA-29c attenuated the inhibitory effect of parecoxib on invasion of U343 cells. Mean±s.d., *n*=3, *P<0.01 compared with U343 cells treated with NC miRNA and PBS; #*P*<0.05 compared with U343 cells treated with NC miRNA and 200 μM of parecoxib.

The effects of miRNA-29c on parecoxib-inhibited invasive ability of U343 cells were further assessed using a Transwell invasion assay. The invasion ability of U343 cells in the miRNA-29c inhibitors group was slightly increased by 21% compared to the NC miRNA group. Moreover, the invasion ability of GBM cells in the miRNA-29c inhibitors+parecoxib group was significantly increased by 26% compared to NC miRNA+parecoxib group (Fig. 7A,B). These results indicate that miRNA-29c inhibitors attenuate the anticancer effects of parecoxib in GBM cells.

## DISCUSSION

In the present study, we found that parecoxib can inhibit cell proliferation, migration and invasion of GBM cells, and identified that miRNA-29c is an important miRNA that is potentially implicated in the inhibitory effect of parecoxib on GBM cell proliferation, migration and invasion.

Cyclooxygenase-2 (COX-2) had been found to be involved in tumorigenesis, and COX-2 inhibitors exhibit a potent anticancer effect (Regulski et al., 2015). As the most famous selective COX-2 inhibitor, celecoxib has highly therapeutic potential for treatment of GBM (Giglio and Levin, 2004), which may be mediated by inhibiting the cell cycle (Kardosh et al., 2004), inducing DNA damage (Kang et al., 2009), and promoting apoptosis (Sareddy et al., 2012) and necrosis (Kang et al., 2007) of GBM cells. Parecoxib is another important selective COX-2 inhibitor that had been clinically used for treatment of postoperative pain with high efficacy and low adverse reaction. Emerging data have indicated that parecoxib can also repress colorectal cancer (Zagani et al., 2009) and esophageal adenocarcinoma (Santander et al., 2012). Moreover, as the prodrug of parecoxib, valdecoxib had been reported to induce apoptosis in esophageal carcinoma Eca109 cells (Liu et al., 2009), inhibit progression of colorectal cancer (Thakral et al., 2010) and reduce breast cancer risk (Ashok et al., 2011). GBM is the most common malignant and deadly brain tumor, and is still among the most difficult tumors to treat despite decades of investigation into therapeutic methods (Young et al., 2014). One limitation for most therapeutic drugs to treat GBM is that the blood–brain barrier can prevent their delivery into the brain (Muldoon et al., 2007). Due to its highly efficient penetration of the blood–brain barrier and potential inhibitory efficacy on multiple tumors, the effect of parecoxib on GBM cells was thus tested. In the present study, we found that treatment with parecoxib significantly suppressed the growth and invasion of GBM cells by upregulating miRNA-29c. Notably, recent studies showed that parecoxib was able to greatly potentiate efficacy of immunotherapeutic treatment of brain tumors (Eberstål et al., 2014, 2012). Based on our and other studies, the clinically available drug parecoxib can not only inhibit the growth, migration and invasion of GBM cells, but also improve immunotherapy of GBM. These data prompted us to further investigate the efficacy of parecoxib on GBM in animal and clinical studies.

miRNA-29c had been found to be significantly downregulated in GBM tissues and cells (Wang et al., 2013; Fan et al., 2013), and forced expression of miRNA-29c significantly inhibits glioma cell proliferation, migration, invasion and angiogenesis (Fan et al., 2013). Consistent with these studies, our data suggested that transfection of miRNA-29c mimics into U251 and U343 cells significantly inhibited cell growth, and transfection of miRNA-29c inhibitors into GBM cells led to attenuation of inhibitory effect of parecoxib on the cell growth, migration and invasion of GBM cells compared to parecoxib treatment alone. Although miRNA-29c was identified as being upregulated by parecoxib to inhibit cell proliferation and invasion abilities of GBM, the mechanisms underlying the anti-tumor effect of parecoxib may be complicated. Firstly, COX-2 expression positively correlates with glioma grade and promotes tumorigenesis (Xu et al., 2014). As a potent and selective COX-2 inhibitor, parecoxib may inhibit transformation and tumorigenesis of GBM cells by directly suppressing COX-2. Secondly, celecoxib can suppress cells proliferation and promote apoptosis in GBM cells by inhibiting the NF-κB signaling pathway (Sareddy et al., 2012), implying that parecoxib may also play the same role in GBM cells by a similar mechanism. These issues will be further defined in future studies.

In conclusion, parecoxib inhibits GBM cell proliferation, migration and invasion by upregulating miRNA-29c. Future studies are required to investigate the roles of parecoxib in GBM in animal models, and further elucidate the mechanisms underlying the anti-tumor effect of parecoxib, which will aid in developing therapeutic strategies for GBM.

## MATERIALS AND METHODS

### Cell culture and parecoxib treatments

Human GBM cell lines including U251 and U343 cells were obtained from the Type Culture Collection of the Chinese Academy of Sciences (Shanghai, China). The GBM cells were cultured in Dulbecco's Modified Eagle's medium (DMEM, Life Technologies, Inc., Carlsbad, CA, USA) supplemented with 10% fetal bovine serum (FBS) (Life Technologies, Inc.), 100 U/ml penicillin and 100 μg/ml streptomycin (Invitrogen, Life Technologies, Inc.), and maintained in a humidified cell incubator at 37°C with 5% CO_2_ and 95% air. Parecoxib sodium (Pfizer Inc., New York, NY, USA) was diluted at concentration of 50 mM, and further diluted in DMEM to obtain the final concentration.

### Cell transfection

100 nM of the scramble miRNA (NC miRNA), miRNA-29c mimics and inhibitors (Genepharma, Inc., Shanghai, China) were transfected into U251 or U343 cells with 70% confluences by using Lipofectamin™ 2000 (Invitrogen, Life Technologies, Inc.) according to manufacturer's instructions. The oligonucleotide sequences of NC miRNA, miRNA-29c mimics and inhibitors are: NC miRNA: 5′-UCACAACCUCCUAGAAAGAGUAGA-3′ (sense), and 5′-UCUACUCUUUCUAGGAGGUUGUGA-3′ (antisense); miRNA-29c mimics: 5′-UAGCACCAUUUGAAAUCGGUUA-3′ (sense) and 5′-UAACCGAUUUCAAAUGGUGCUA-3′ (antisense); miRNA-29c inhibitors: 5′-UAACCGAUUUCAAAUGGUGCUA-3′.

### MTT assay

After treatment, the cell growth of U251 and U343 cells plated in 96-well plates was detected by using 3-(4,5-dimethylthiazol-2-yl)-2,5-diphenyltetrazolium bromide (MTT) assay. Briefly, the cells were treated with MTT and incubated at 37°C for 4 h. Supernatant was removed and the dye was dissolved with 100 μl of DMSO and shaken on an orbital shaker for 10 min in the dark. The optical density (OD) was recorded at 570 nm using a spectrophotometer (Bio-Rad, Hercules, CA, USA). Each experiment was performed in triplicate.

### BrdU incorporation assay

DNA synthesis in proliferating U251 and U343 cells was determined by using 5-Bromo-2-deoxyUridine (BrdU) incorporation assay. 10 μM of BrdU (BD Pharmingen, San Diego, CA, USA) was added to the cells for 1 h. Cells were washed with PBS and fixed for 20 min in 4% paraformaldehyde (PFA). After blocking with 10% goat serum for 1 h, cells were incubated with peroxidase-coupled anti-BrdU-antibody (1: 200, Abcam) overnight at 4°C, washed three times with PBS, subsequently incubated with peroxidase substrate (tetramethylbenzidine) for 1 min at room temperature. The absorbance at 450 nm was determined with a spectrophotometer. Each experiment was performed in triplicate.

### RNA extraction and quantitative real-time PCR (RT-qPCR)

Total RNA was extracted from U251 and U343 cells with TRIzol reagent (Life Technologies, Inc.), and 100 ng total RNA was subsequently reverse transcribed into cDNA by using TaqMan MicroRNA Reverse Transcription Kit (Applied Biosystems, Carlsbad, CA, USA) and mRNA Reverse Transcription Kit (Takara, Dalian, Liaoning, China) according to the manufacturer's protocol. RT-qPCR analysis was performed in the 7500 RT-qPCR system (Applied Biosystems) using the SYBR Green PCR Master Mix (Applied Biosystems) under the following conditions: DNA denaturation at 95°C for 10 min, followed by 40 cycles of 95°C, 15 s for denaturation, 60°C, 60 s for annealing and extension. Small nuclear U6 snRNA (Applied Biosystems) was used as the endogenous control to miRNA-29c. The RNA levels were determined using the 2^−ΔΔCt^ method. All primers were purchased from GeneCopoeia (Guangzhou, Guangdong, China). All experiments were conducted in triplicate.

### Wound-healing assay

After treatment, U343 cells in six-well plates were scratched with a sterile 10 μl pipette tip three times to form parallel lines, subsequently washed by PBS for removal of non-adherent cells. The six-well plates were then incu­bated at 37°C, 5% CO_2_ for 24 and 48 h, and the same wound areas were observed and photographed under an inverted microscope (Olympus CKX41, MA, USA). The distance of the scratch closure was examined at 0, 24 and 48 h.

### Cell invasion assays

The transwell assays were performed in 24-well chambers (Corning Incorporated, Corning, NY, USA) to detect the effect of miRNA-29c mimics and inhibitors on the invasion of U343 cells. After transfection with miRNA-29c mimics or inhibitors, U343 cells were trypsinized, washed and resuspended in DMEM medium with 1% FBS, and seeded into the upper chamber of the wells with the density of 1×10^4^. Simultaneously, 400 μl of DMEM medium with 10% FBS was placed in the lower chamber in a 24-well cultural plate. After cells were cultured for 24 h at 37°C in 5% CO_2_, non-invading cells were removed with cotton swabs, and invaded cells on the bottom surface of filter were fixed in 100% methanol and stained with 0.1% Crystal Violet. Subsequently, the stained cells in the filter were photographed and counted under an inverted microscope at ×400 magnification from 10 random fields. Assays were performed in triplicate.

### Statistical analysis

All statistical analyses were carried out by using SPSS 18.0 software (IBM). Results are expressed as mean±s.d. from the experiments that repeated at least three times, Student's *t*-test or one-way ANOVA analysis were used to compare differences between groups. *P*-values <0.05 were considered statistically significant.
